# Variability of Postural Stability and Plantar Pressure Parameters in Healthy Subjects Evaluated by a Novel Pressure Plate

**DOI:** 10.3390/ijerph19052913

**Published:** 2022-03-02

**Authors:** Allegra Fullin, Paolo Caravaggi, Pietro Picerno, Massimiliano Mosca, Silvio Caravelli, Antonio De Luca, Angela Lucariello, Paolo De Blasiis

**Affiliations:** 1 Department of Mental and Physical Health and Preventive Medicine, Section of Human Anatomy, University of Campania “Luigi Vanvitelli”, 80138 Naples, Italy; allegrafullin92@hotmail.it (A.F.); antonio.deluca@unicampania.it (A.D.L.); 2 Movement Analysis Laboratory, IRCCS Istituto Ortopedico Rizzoli, 40136 Bologna, Italy; paolo.caravaggi@ior.it; 3 SMART Engineering Solutions & Technologies (SMARTEST) Research Center, Università telematica “e Campus”, 22060 Novedrate, Italy; pietro.picerno@uniecampus.it; 4 II Clinic of Orthopaedics and Traumatology, IRCCS Istituto Ortopedico Rizzoli, 40136 Bologna, Italy; massimiliano.mosca@ior.it (M.M.); doct.car@gmail.com (S.C.); 5 Department of Sport Sciences and Wellness, University of Naples “Parthenope”, 80100 Naples, Italy; angela.lucariello@gmail.com

**Keywords:** postural stability, posture, plantar pressure, pressure plate, accuracy, repeatability, baropodometry, stabilometry

## Abstract

Background: Postural stability and plantar pressure parameters can be assessed by baropodometry; nevertheless, they are often affected by low repeatability. The aim of the study was to test the accuracy and repeatability of a novel resistive sensor pressure plate and to establish the most reliable baropodometric parameters. Methods: Accuracy and repeatability of the FM12050 BTS-Bioengineering plate measurements were assessed by using different weights in static conditions across three sessions. Subsequently, 20 healthy subjects were assessed by 30-s stabilometric analysis in bipedal standing with open eyes across four trials in two sessions, morning and afternoon. Results: Pressure plate repeatability in measuring the static weights was very high, and plate measurements were correlated to the scale measurements (Pearson’s coefficient = 0.99). Percentage of load distribution between left and right foot and in rearfoot and forefoot regions showed the largest repeatability (coefficient of variation < 5%) across trials. Eventually, median and percentiles (25–75%) were reported for each parameter. Conclusions: This study helped to assess the accuracy and repeatability of a novel pressure plate in static conditions and to define the most reliable parameters for the assessment of postural stability and foot morphology. The present healthy-subject stabilometric dataset may be used as reference data in the evaluation of pathological populations.

## 1. Introduction

Upright posture is maintained by tonic muscle contractions stabilizing the positions of body segments against the force of gravity [1]. The Postural Control System (PCS), consisting of key brain nodes (brainstem, cerebellum, basal ganglium, thalamus, and several cortical regions) [2] and peripheral receptors (visual, stomatognathic, vestibular, and somatosensory), is responsible for postural control, stability, and balance [2,3]. The PCS ensures minimal energy expenditure through functional neuromuscular adaptation and biomechanical strategies, which maintain the vertical projection of the center of body mass (CoM) within the feet support area [2]. Since the CoM is located on the trunk, and the base of support is on the ground, the posture is inherently unstable, and the body CoM dynamics can be compared to an “inverted pendulum” oscillating around the ankle joint in a sway cone of about 4 degrees (°) [4,5]. CoM is defined as the point where body mass can be assumed to be concentrated in a three-dimensional (3D) body, whereas the center of pressure (CoP) is the instantaneous position of vertical ground reaction force on the ground plane [6]. Postural stability concerns the ability to control the body position in the space [7] and is critical to maintain upright posture by minimizing the oscillations of the CoP. Postural stability and the body oscillations can be assessed quantitatively by stabilometric examination using a pressure plate [6]. CoP is calculated as the centroid of the total number of active sensors for each data sample collected, which represents the spatial distribution of pressure over time [6]. Body sway can be quantified by the ellipse circumscribing the CoP trajectory recorded during the exam, defined as the center-of-pressure sway area (CoPsa). Moreover, other parameters can be determined, such as the length surface function (LSF, i.e., the ratio between distance covered by the CoP during a standing trial and CoPsa [8]) and the speed of the center of pressure (CoP speed, computed from the instantaneous CoP displacement [9]). Additionally, the pressure plate is also able to assess the foot-to-ground load distribution by mapping the pressure in different plantar regions in static and dynamic conditions, with a spatial resolution equal to the pressure sensor surface. The pressure distribution between two feet and, for each foot, between different plantar regions can also be assessed. Finally, the arch index (AI), that is, the ratio between midfoot contact area and foot contact area (which does not consider the toes’ pressure), can be also evaluated and used to classify a foot typology (normal arched, cavus, or flat). The two main technologies used for the plantar pressure sensors are capacitive and resistive: while the former exhibit low hysteresis and temperature sensitivity and a higher repeatability of measurements, the latter are less expensive and thus mostly used in the clinical practice. Although these devices are commonly used to investigate the foot-to-ground dynamic interaction (albeit only in an orthogonal direction to the plate) with a spatial resolution higher than that of force plates, pressure measurements are often affected by low repeatability. This is mainly due to the technology type, size and number of sensors, the sampling frequency, and a lack of standardized calibration procedures [10,11]. Understanding the accuracy and repeatability of the instrumentations is critical for the correct interpretation of measurements in clinical and research studies [12]. In addition, the large number of pressure devices and software available has so far prevented to establish a reliable reference dataset of pressure-based parameters in common motor tasks. Finally, pressure plate users should be able to interpret the instrument output concerning the instrument’s accuracy and repeatability. The accuracy and the repeatability of pressure plates used in clinical settings have been previously investigated [13]. In particular, several studies have analysed the intra-subject variability of stabilometric and/or plantar pressure parameters during the same day in healthy subjects [14,15,16] or sportsman [17]. A few studies assessed the variability of these parameters in a young, healthy population during semi-static and dynamic tests in two sessions one week apart [18] or intra-session and inter-session over two weeks [19]. Other studies assessed the repeatability and the reliability of novel pressure plates [20,21,22]. Finally, two studies defined the standard reference values of the postural control and pressure in the static condition as median [23] and as mean values [24], respectively. To the best of the authors’ knowledge, no previous study analysed P-walk (FM12050 BTS-Bioengineering) resistive pressure plate and the intra-subject variability of plantar pressure and postural stability parameters in healthy subjects within the same day. The aim of this study was, hence, twofold: to test the accuracy and the repeatability of this novel pressure plate based on resistive-sensor technology and to establish, for this instrumentation, a set of reliable baropodometric parameters assessed during bipedal upright posture in a healthy population.

## 2. Method and Materials

**Figure 1:**
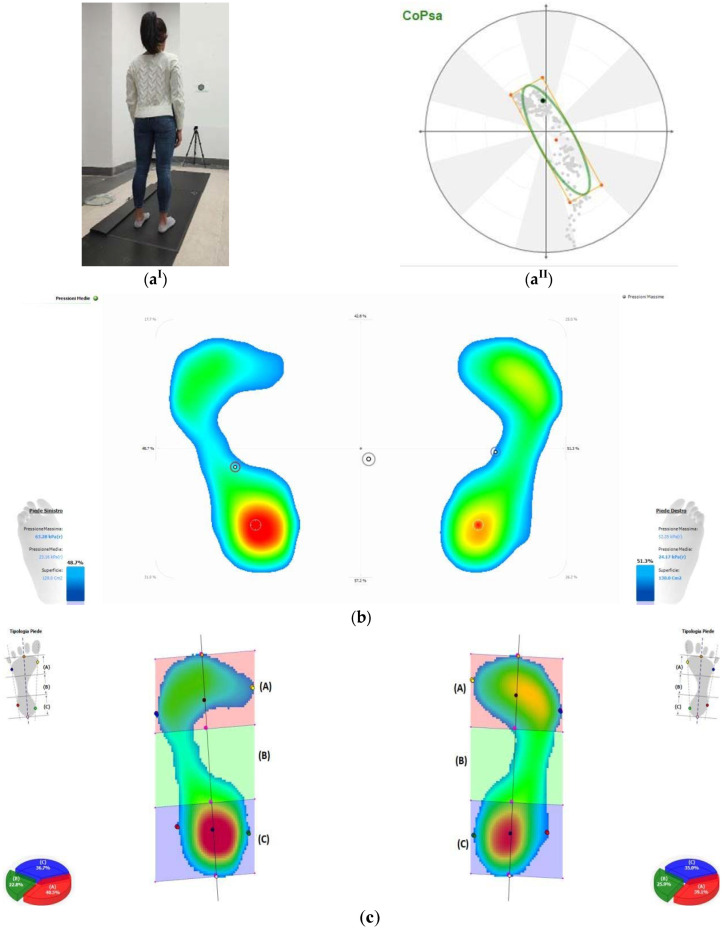
Subject in anatomical standing posture (**a^I^**), stabilometric (**a^II^**), and plantar pressure (**b**,**c**) parameters. (**a**) (CoPsa, center of pressure sway area); (**b**) (percentage of load distribution); (**c**) (division of foot in three equal regions: forefoot, A; midfoot, B; rearfoot, C).

The repeatability and accuracy of a new 200 × 50 cm 10.000 sensors/m2 pressure plate (P-Walk FM12050 BTS-Bioengineering, Milan, Italy) were evaluated at the Functional Anatomy Laboratory of the University of Campania “L. Vanvitelli”. Static baropodometric examinations were performed by placing two plastic containers (17.8 × 13.2 × 39.6 cm^3^; 0.26 kg) on the pressure plate to simulate a bi-podalic standing condition. Two plexiglass plates (20 × 1 × 40 cm^3^; 0.92 kg) were placed between the containers and the pressure plate to obtain a uniform load distribution. The static tests were performed using three different weights by filling each of the two containers with 4 L, 5 L, and 6 L of water for a total weight of 101.6, 121.3, and 140.9 N. Each static trial lasted 30 s, and it was performed three times across three sessions in three different days. After each test, the containers were removed from the plate, and a weight-calibration procedure was performed. A pressure matrix (a × b × n, where a and b are the numbers of sensors making the pressure image, and n is the number of frames recorded) was exported for each static trial. The total load estimated by the plate in each trial was calculated as the sum of the product between each sensor pressure by its surface (1 cm2) and averaged across all frames. To assess accuracy and repeatability of the measurements, weights of containers estimated by the pressure plate were compared to the same ones measured by a precision scale (Mod. MV-F-S). Bland–Altman plots were used to analyse the pressure plate accuracy in measuring the static weights across trials. To assess the inter-trial and intra-subject variability of the stabilometric parameters, 20 healthy subjects (7 males, 13 females; age 20.2 ± 0.9 years; right-handed 20/20; height = 1.69 ± 0.09 m; weight = 61.94 ± 8.58 kg; BMI = 21.6 ± 1.7 kg/m^2^) were recruited, and all of them gave written consent for participation. Ethical review and approval were waived for this study due to the nature of this pilot study, which required the recruitment of a small population of healthy participants tested for standard baropodometric parameters. The following inclusion criteria were used: absence of pain; no surgeries in the last 6 months; no muscle-skeletal injuries in the last 3 months; no dental surgery or use of dental implants; no prostheses or use of corrective orthoses; no neurological or visual disease; no skeletal dysmorphism; and no cognitive impairment. Participants were evaluated in upright bipedal posture on the pressure plate (P-Walk FM12050 BTS-Bioengineering), sampling at 50 Hz, following the international standardization criteria for baropodometric tests [25]: anatomical standing posture with the arms relaxed along the body close to the thighs, the head in neutral position, with open eye, and the visual target positioned 2 m away, (except for the feet placement, maintained in a usual comfortable position and at the same distances between heels and toes for each trial; Figure 1a^I^). To ensure a correct postural examination, the tests were performed in silence in a room with a level floor and white walls. Four stabilometric trials of 30 s each were performed in two sessions (morning and afternoon).

The following clinically relevant stabilometric and pressure parameters were measured: center-of-pressure sway area (CoPsa), the length surface function (LSF), center-of-pressure speed (CP speed), foot load; rearfoot (Rf) load, midfoot (Mf) load, forefoot (Ff) load (Figure 1a^II^,b,c), mean (Pmean), and maximum pressure (Pmax). Load parameters were normalized to body weight (%BW). The division of the foot in three equal areas (Rf, Mf, and Ff) was calculated by software considering the total area defined by the most extreme points on the four sides of the foot. The ratio between midfoot contact area and whole foot contact area, known as the arch index, was also estimated. Mean and standard deviation (SD) or median and percentiles (25% and 75%) were used to report data, respectively, for parametric or non-parametric distributions. Variability of all the stabilometric parameters across trials in open-eyes (OE) condition was assessed via coefficient of variation (CV). The most reliable pressure and postural stability parameters were identified. Statistical differences in each baropodometric and stabilometric parameter between sessions were assessed via Pearson’s coefficient of correlation (R^2^). Statistical analysis was performed using Matlab (Mathworks) and R [26].

## 3. Results

### 3.1. Repeatability and Accuracy of Pressure Plate Static Measurements

**Figure 2:**
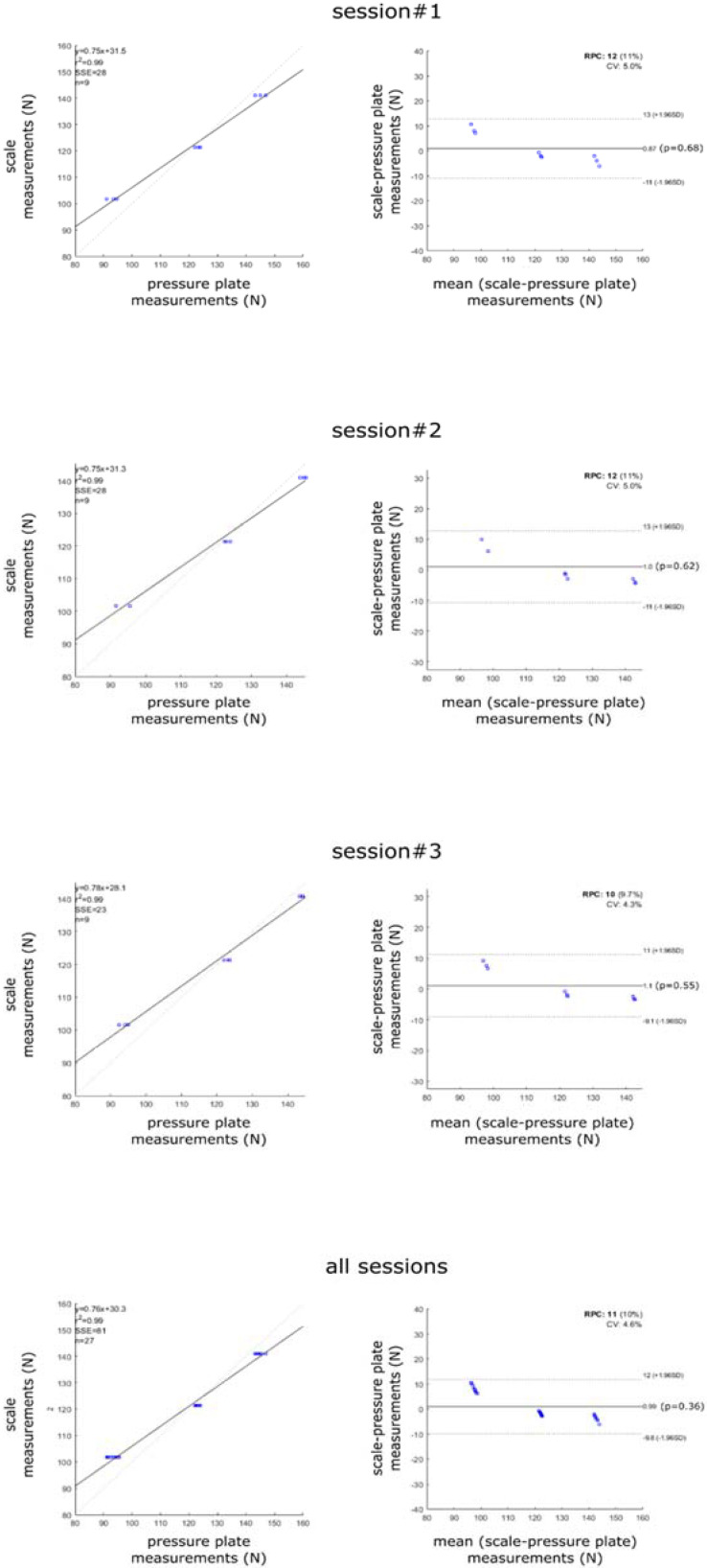
Bland–Altman plots for each of the three measurements’ sessions (**top**), and of all measurements across all sessions (**bottom**). Correlation coefficient (R^2^); sum of square error (SSE), variation coefficient (CV), standard deviation (SD).

Pressure plate repeatability in measuring the static weights was high. The coefficient of variation ranged between 0.014–0.025 (1.4–2.5%) for the smallest weight, between 0.007–0.008 for the intermediate weight, and between 0.035- 0.014 for the largest weight across the three sessions. Bland–Altman plots report the error in measuring each of the three weights in the three sessions (Figure 2). Across sessions, the mean error ranged between 0.9 and 1.1 N, the confidence interval (CI) lower bound between −9.1 and −11.0 N, and the CI upper bound between 11.0 and 13.0 N. Plate measurements were highly correlated to the scale measurements (R^2^ = 0.99).

### 3.2. Intra-Subject Variability Test for Baropodometric and Stabilometric Parameters

**Figure 3:**
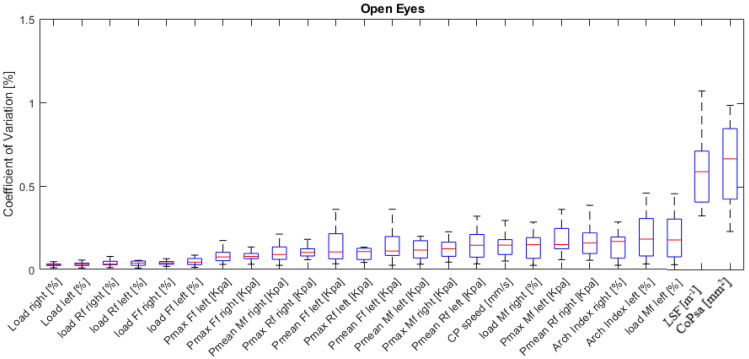
Boxplot of inter-subject coefficient of variation (%) of stabilometric and pressure parameters sorted in ascending order to median values. Forefoot (Ff); midfoot (Mf); rearfoot (Rf); mean pressure (Pmean); maximum pressure (Pmax); center-of-pressure speed (CP speed); length surface function (LSF); center-of-pressure sway area (CoPsa).

The boxplots (median 25–75%) of the inter-subject CV report the variability of stabilometric parameters across trials (Figure 3). Parameters’ variability was sorted in ascending order by the median value. The percentage of load distribution between left and right foot and in rearfoot and forefoot regions showed the lowest variability (CV < 5%). Mean and peak pressure parameters, along with the load on the midfoot regions, showed slightly larger variability (CV ~ 5–15%). The largest variability was found for CoPsa and LSF (CV > 50%).

### 3.3. Inter-Session Variability Test (Morning vs. Afternoon)

Table 1 shows the inter-session Pearson (R^2^) for each baropodometric and stabilometric parameter. No statistically significant correlation (*p* > 0.05) was found for left and right load distributions, for CoPsa, and for LSF. The largest correlations (R^2^ > 0.85) were found for Rf, Mf, and Ff loads and for AI.

### 3.4. Normative Stabilometric and Baropodometric Parameters

Table 2 reports the inter-subject median (25–75%) of each stabilometric and plantar pressure parameter. Foot load percentage was larger on the right side and, on each side, was larger on the Ff than on the Mf and Rf. As predictable, the midfoot was the least loaded foot region and showed the lowest contact region. With respect to the pressure parameters, the largest P-mean was observed on the Ff and the largest Pmax on the Rf.

## 4. Discussion

Understanding the intra- and inter-subject variability of the parameters in common postural testing conditions is critical for the interpretation of measurements and to determine the correct sample size to ensure sufficient statistical power in clinical and research investigations. For the variety of pressure platforms and relevant software available, a dataset of the more reliable stabilometric and plantar pressure parameters in controlled testing conditions should be collected and used as a reference for the device used in a specific laboratory. Similar to what was performed in other studies [20,21], this study is concerned with the evaluation of a novel pressure plate based on resistive sensors. The plate has been assessed in terms of accuracy in measuring static loads and for repeatability in measuring standard stabilometric and plantar pressure parameters during bipedal standing with open eyes in anatomical position in a population of young, healthy subjects. The degree of agreement between pressure-based measurements of load and the actual weight applied to the plate, i.e., the accuracy in static testing conditions, was good (max errors < 10%; Figure 2). It should be highlighted that each accuracy trial was preceded by the weight calibration of the pressure plate following the manufacturer’s instructions. In terms of repeatability of stabilometric and plantar pressure measurements, CV was lower than 15% for most parameters except CoPsa and LSF (Figure 3). These baropodometric parameters, recorded in healthy young subjects, presented high repeatability across trials following that reported by [15,16,20] even though a significant intra-subject variability of CoP speed was also reported in the same day by [22]. Moreover, in [18], high reliability measured across two sessions one week apart was also observed (ICC ≥ 0.70) for AI, Pmean, Ff, and Mf loads but also for CoPsa, partly in agreement with our study. Another study [14] reported the CoPsa as reliable measure, too, in contrast with our study. Instead, a lowest repeatability was observed for LSF and CoPsa (CV > 50%) [19] in agreement with our study. The extension of the boxplots for these two parameters also shows a large inter-subject variation of CV (Figure 3). Thus, attention should be paid when interpreting these data and in the assessment of differences between groups. Concerning the pressure measurements, results of our study showed slightly larger variability (CV ~ 5–15%) for Pmean and Pmax as reported in [21], but these findings disagreed with [14], where pressure parameters obtained low reliability, and in particular, Pmax showed lower reliability than Pmean. Inter-session repeatability between morning and afternoon sessions was high for most parameters, above all for Rf, Mf, and Ff load percentages and arch index in both sides (R^2^ > 0.85) as reported in Table 1. About normal values (Table 2), the current sample of subjects’ feet could be classified as normal-arched in the right side (median = 0.21) and a mild cavus feet in the left side (median = 0.14) according to the classification proposed by Cavanagh and Rodgers [27] (AI of normal-arched feet: 0.21 < AI < 0.26 albeit with a different measuring system). This result is consistent with a larger median load observed on the right foot (53.5%) with respect to the left one, in agreement with [23]; in fact, a greater load may explain a greater biomechanical adaptation of the medial longitudinal arch with a trend of decrease of cavus feet [28]. Moreover, on each side, a larger load on the Ff (43%) with respect to the Mf and Rf was found in accordance with [18]. Previous studies [23,24,29,30] analysed Rf and Ff data exclusively, and with this subdivision, a largest Rf load was found. Eventually, on both feet, a larger Pmax on the Rf area (left = 67.3 KPa; right = 63.3 KPa) with respect to the Ff one was found in agreement with [29], while a larger Pmean in Ff with respect to other two regions was found with a median value of 59.1 KPa on the left side and 69.9 KPa on the right one. Finally, normal median values of CoPsa (28.8 mm2), LSF (4.7 m−1), and CP speed (3.4 mm/s) in OE condition confirmed the normal 244 range values found in [30,31,32], respectively, in healthy, young subjects.

While these normal data (Table 2) are rather consistent across the healthy subjects recruited in the present study and thus have general validity outside the scope of this paper, these should be used with caution if used to compare data acquired with different instrumentation or in different laboratory conditions. A limitation of this study is the small sample size because of strict inclusion criteria and, in particular, for visual and dental impairments. Moreover, the pressure plate was weight calibrated before each measurement following the procedure indicated by the manufacturer. While this procedure has guaranteed high accuracy of the pressure recorded by each sensor, the authors understand that, in clinical gait labs, instruments calibration is normally performed once a day, and thus, lower accuracy may be expected in standard working conditions. In addition, due to the chosen testing setup, only rather low weights could be applied to the device (<150 N). Accuracy should be estimated by applying larger loads more consistent with the average body weight (700 N), as this may vary significantly for larger loads. The reference dataset of stabilometric and plantar pressure parameters for bipodalic open-eyes standing posture, established for the present pressure platform, may be useful for the assessment of pathological populations or evaluation of the same subject during stimuli of different posture receptors.

## 5. Conclusions

This study helped to assess the accuracy and repeatability of a novel, resistive-sensor pressure plate in controlled laboratory conditions. Most stabilometric and plantar pressure parameters showed low variability during multiple trials conducted on the same day. A reference dataset of baropodometric and stabilometric parameters for open-eyes bipodalic standing posture was collected and reported. These data may be useful to clinical evaluation for detecting postural disorders or adaptations to different stimuli of postural receptors.

## Figures and Tables

**Table 1:** Inter-session repeatability of measurements between the two sessions via Pearson’s (R^2^) correlation coefficient for each parameter. NS indicates correlations not statistically significant (*p* < 0.05). Statistically significant correlations (R^2^ > 0.70) are highlighted in bold. Not significant (NS); forefoot (Ff); midfoot (Mf); rearfoot (Rf); mean pressure (Pmean); maximum pressure (Pmax); center-of-pressure sway area (CoPsa); center-of-pressure speed (CP speed); length surface function (LSF).

Stabilometric and Plantar Pressure Parameters	Pearson R^2^
Load left (%)	NS
Load right (%)	NS
CoPsa (mm^2^)	0.37
CP speed (mm/s)	NS
Load Rf left (%)	**0.85**
Load Rf right (%)	**0.86**
Load Mf left (%)	**0.91**
Load Mf right (%)	**0.89**
Load Ff left (%)	**0.76**
Load Ff right (%)	**0.87**
Arch Index right (%)	**0.88**
Arch Index left (%)	**0.91**
LSF (mm^−1^)	NS
Pmean Ff left (Kpa)	0.55
Pmean Mf left (Kpa)	0.46
Pmean Rf left (Kpa)	0.66
Pmean Ff left (Kpa)	0.51
Pmean Mf right (Kpa)	0.23
Pmean Rf right (Kpa)	0.67
Pmax Ff left (Kpa)	0.71
Pmax Mf left (Kpa)	NS
Pmax Rf left (Kpa)	0.62
Pmax Ff right (Kpa)	0.62
Pmax Mf right (Kpa)	NS
Pmax Rf right (Kpa)	0.57

**Table 2:** Normative values for all baropodometric parameters reported as inter-subject median (25% 75%). Not significant (NS); forefoot (Ff); midfoot (Mf); rearfoot (Rf); mean pressure (Pmean); maximum pressure (Pmax); center-of-pressure speed (CP speed); center-of-pressure sway area (CoPsa); length surface function (LSF).

Stabilometric and Plantar Pressure Parameters		Percentiles	
	Median	25%	75%
Load left (%)	46.5	45.7	48.7
Load right (%)	53.5	51.3	54.4
CoPsa (mm^2^)	28.8	23.5	41.7
CP speed (mm/s)	3.4	3.1	3.9
Load Rf left (%)	41.2	36.5	44.9
Load Rf right (%)	36.9	33.9	42.0
Load Mf left (%)	14.1	8.6	25.1
Load Mf right (%)	20.8	12.9	24.9
Load Ff left (%)	43.9	41.0	45.1
Load Ff right (%)	43.1	40.9	43.9
Arch Index right (%)	20.6	12.9	24.9
Arch Index left (%)	14.1	8.6	25.3
LSF (mm^−1^)	4.7	3.8	7.1
Pmean Ff left (Kpa)	59.1	53.2	67.9
Pmean Mf left (Kpa)	16.4	15.0	18.8
Pmean Rf left (Kpa)	27.4	23.8	35.6
Pmean Ff left (Kpa)	69.9	60.3	83.9
Pmean Mf right (Kpa)	18.3	17.0	20.0
Pmean Rf right (Kpa)	23.2	18.7	31.0
Pmax Ff left (Kpa)	44.5	41.8	49.3
Pmax Mf left (Kpa)	32.1	27.3	38.8
Pmax Rf left (Kpa)	67.3	59.5	80.5
Pmax Ff right (Kpa)	49.6	48.3	55.0
Pmax Mf right (Kpa)	35.9	33.3	38.3
Pmax Rf right (Kpa)	63.3	55.8	71.0

## Data Availability

Not applicable.
